# Hip Labral Morphological Changes in Patients with Femoroacetabular Impingement Speed Up the Onset of Early Osteoarthritis

**DOI:** 10.1007/s00223-023-01076-1

**Published:** 2023-03-22

**Authors:** Michela Battistelli, Enrico Tassinari, Giovanni Trisolino, Marco Govoni, Gianluca Ruspaggiari, Lucia De Franceschi, Dante Dallari, Debora Burini, Roberta Ramonda, Marta Favero, Francesco Traina, Brunella Grigolo, Eleonora Olivotto

**Affiliations:** 1 Department of Biomolecular Science, Urbino University “Carlo Bo”, Via Ca’ Le Suore 2, 61029 Urbino, PU Italy; 2 Orthopaedic-Traumatology and Prosthetic Surgery and Revisions of Hip and Knee, IRCCS Istituto Ortopedico Rizzoli, Bologna, Italy; 3 Pediatric Orthopedic and Traumatology, IRCCS Istituto Ortopedico Rizzoli, Bologna, Italy; 4 Reconstructive Orthopedic Surgery and Innovative Techniques - Musculoskeletal Tissue Bank, IRCCS Istituto Ortopedico Rizzoli, Bologna, Italy; 5 Department of Medicine (DIMED), Rheumatology Unit, University Hospital of Padova, Padua, Italy; 6 Medicine Unit 1, Ca’ Foncello Hospital, Treviso, Italy; 7 RAMSES Laboratory, RIT Department, IRCCS Istituto Ortopedico Rizzoli, Bologna, Italy

**Keywords:** Labrum, Femoroacetabular impingement, Osteoarthritis, Extracellular matrix degeneration, Calcification

## Abstract

Over the last decade, evidence has mounted for a prominent etiologic role of femoroacetabular impingement (FAI) in the development of early hip osteoarthritis (OA). The aim of this study was to compare the ultrastructure and tissue composition of the hip labrum in healthy and pathological conditions, as FAI and OA, to provide understanding of structural changes which might be helpful in the future to design targeted therapies and improve treatment indications. We analyzed labral tissue samples from five healthy multi-organ donors (MCDs) (median age, 38 years), five FAI patients (median age, 37 years) and five late-stage OA patients undergoing total hip replacement (median age, 56 years). We evaluated morpho-functional by histology and transmission electron microscopy. Extracellular matrix (ECM) structure changes were similar in specimens from FAI compared to those from patients with OA (more severe in the latter) showing disorganization of collagen fibers and increased proteoglycan content. In FAI and in OA nuclei the chromatin was condensed, organelle degenerated and cytoplasm vacuolized. Areas of calcification were mainly observed in FAI and OA labrum, as well as apoptotic-like features. We showed that labral tissue of patients with FAI had similar pathological alterations of tissue obtained from OA patients, suggesting that FAI patients might have high susceptibility to develop OA.

**Supplementary Information:**

The online version contains supplementary material available at 10.1007/s00223-023-01076-1.

## Introduction

Hip Osteoarthritis (HOA) is the most common joint disorder of the hip and a major cause of disability in the adult population [1, 2]. Thus, the early diagnosis, prevention and treatment of the early stages of the disease and of the pre-arthritic condition, especially in adolescents and young adults, is crucial to reducing the incidence of end-stage HOA and the need for total hip replacement (THR).

The = femoroacetabular impingement (FAI) represents the most common mechanism that leads to the development of early cartilage and labral damage in the non-dysplastic hip [3]. There are two types of FAI, one caused by an abnormality in acetabular shape or orientation (pincer), and the other by a shape abnormality in the proximal femur (cam). Frequently, a combined impingement occurs when both the pincer and cam types are present. In pincer impingement, the abnormal contact between the femoral neck and the over-covered acetabular rim is thought to cause an impaction type of impingement, which compresses the labrum, and could subsequently lead to circumferential cartilage damage. The cam type is caused by extra bone formation at the anterolateral head-neck junction, which results in a non-spherical, cam-shaped abnormality. The cam is forced into the acetabulum during flexion and internal rotation of the hip and the impingement can cause an inclusion type of damage to the acetabular labrum and articular cartilage, which is typically accompanied by detachment of the acetabular cartilage from the subchondral bone [4].

Considerable evidence has mounted supporting a prominent etiologic role of FAI in the development of early HOA in middle-aged adults in particular Ganz et al. described the FAI role in OA development [5].

FAI is associated with chondro-labral damage in young asymptomatic adults [6], and it may lead to hip pain in asymptomatic young individuals [7]. The role of cam FAI on the development of end-stage OA has been demonstrated in longitudinal studies from two large, population-based cohorts The presence of cam deformities at baseline substantially increases the risk of developing OA and the need for THR in middle-aged adults (the positive predictive value for the development of hip OA is estimated at 6–25%, with follow-up times of 5–19 years) [4, 8]. A cam FAI morphology is recognizable in more than 50% of cases of premature OA [9, 10], as discussed by Casartelli et al. in a recent review [11], cam morphology and hip dysplasia are associated with hip OA as supported by both prospective and cross-sectional studies.

Moreover, in our previous study we described the presence of joint tissue degeneration in patients with FAI, in particular labral tears were present in 86% of patients and labral calcium crystal deposition in 67% of them [12]. We also found macroscopic cartilage lesions in 90% of patients, predominantly at the acetabular side. The hip labrum is a fibrocartilaginous structure that provides joint stability and is involved in load distribution [13]. Labral deterioration or tear leads to impairment of its functions and changes the hips biomechanics. Prior work has demonstrated that impaired lubrication and an increased joint friction deteriorate articular cartilage and lead to early OA [14].

The aim of the present study was to investigate the extracellular matrix (ECM) composition and cell morphology of normal, injured, and OA labrum of the human hip, by histological analysis and Transmission Electron Microscopy (TEM), in order to understand structural changes suitable in the future to design targeted therapies and to improve treatment indications.

## Materials and Methods

### Patient Recruitment, Clinical Data and Sample Collection

This observational study was conducted in accordance with the approval of the Local Ethical Committees (EM711-2019_94/2013/Oss/IOR_EM2). Five patients with FAI undergoing arthroscopy at the Orthopaedic-Traumatology and Prosthetic Surgery and Revisions of Hip and Knee (IRCCS Istituto Ortopedico Rizzoli, Bologna, Italy) were enrolled after providing informed consent (two males and three females; median age: 36.8 years; interquartile range [IQR]: 28–51; median body mass index (BMI): 24.6 kg/m2, IQR: 19.2–31. See Supplementary Table 1 for patient details.

**Fig. 1:**
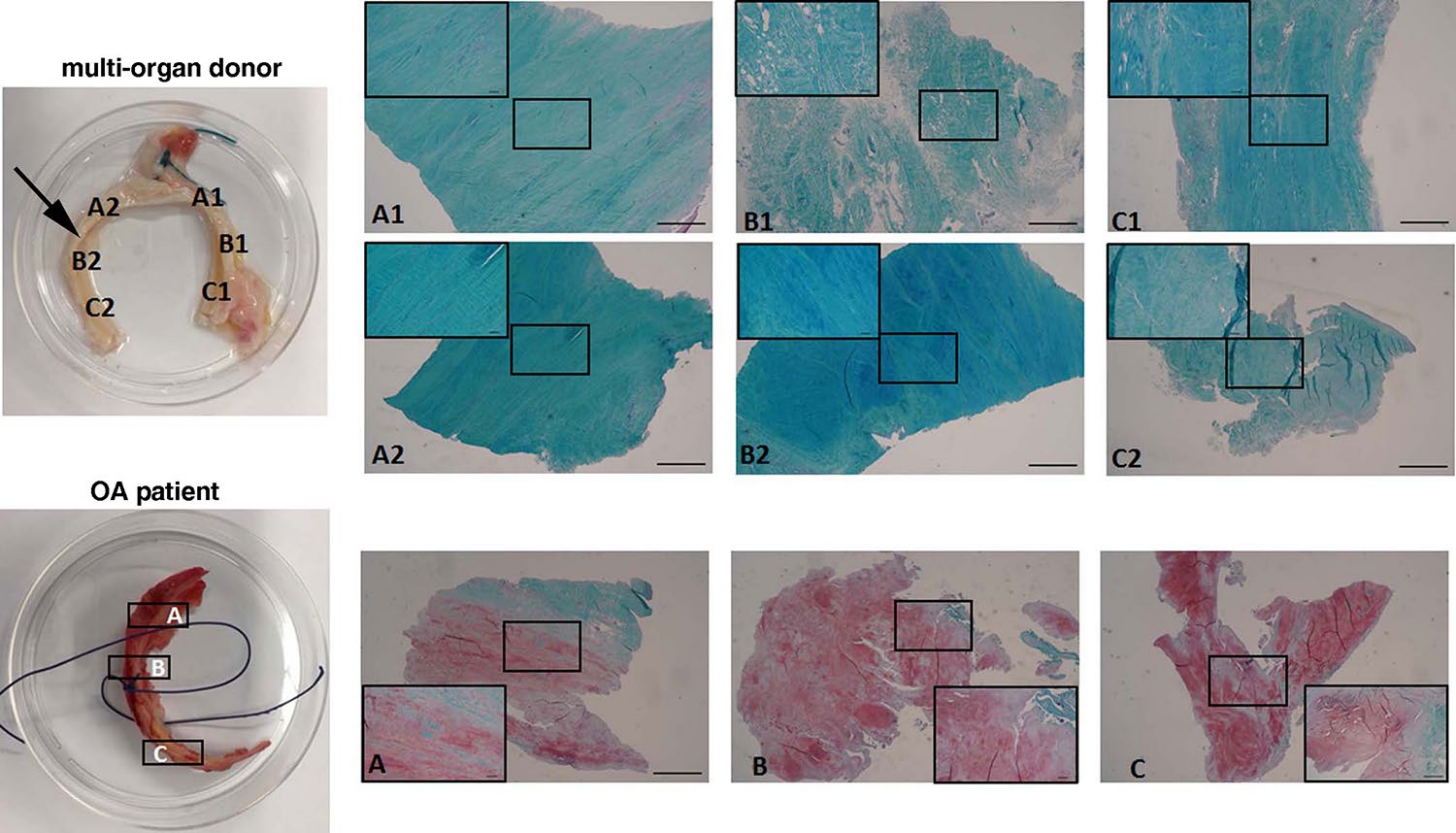
Labral tissue samples. Upper side: samples from one representative multi-organ donor (the arrow indicates the area where the biopsies were collected in FAI patients). Lower side: samples from one representative patient with end-stage OA. For both samples, LEFT: high resolution digital photograph of labral tissue; RIGHT: Safranin-O-Fast Green matrix staining for the histological grading score. A = anterior, B = middle and C = posterior region of each labral sample cut perpendicular relative to the sagittal plane (Magnification 2x, bar 1000 μm; insert 10x, bar 100 μm)

These patients had initial diagnosis of cam FAI in two cases, pincer FAI in one case and mixed FAI in two cases. The frayed labral tissue in the area of impingement, that would be excised and otherwise discarded, was retrieved for histological analysis. Labral tissue from patients with end-stage OA, classified with Kellgren and Lawrence (KL) system grade four [12], were harvested from five patients undergoing THR (two males and three females, median age 55.6 years with IQR of 45–64; median BMI 27.5 kg/m2 with IQR of 24.4–30.7) (Supplementary Fig. 1). As control, the labrum from five multi-organ cadaver donors (MCDs) without a history of joint disorders (five males, median age 37.8 years with IQR of 17–48; median BMI 24.9 kg/m2 with IQR of 20.1–26.3) was collected by the Musculoskeletal Tissue Bank of IRCCS Istituto Ortopedico Rizzoli (Bologna, Italy; EU TE Code: IT000096) during its routinely activities of tissue procurement, following the National Transplant Centre (CNT) [15] and European Directorate for the Quality of Medicines & HealthCare (EDQM) guidelines [16, 17]. Specifically, only the residual labral tissue i.e., a portion considered as a waste material remained attached to epiphysis during femur procurement procedures was used for the histo-morphological analyses. Because the acetabulum is a spherical structure, the topographical structures within and around it has conventionally been described according to a clock-face method [18, 19], considering the midpoint of the transverse acetabular ligament as the 6:00 position (left hip). In our study in samples from MCDs and OA patients were marked on the femoral sides with a stitch to maintain orientation during analysis. Two consecutive slices, one processed for histology and one for TEM, were resected from the entire tissue sample (total three different tissue samples for half labrum or six for the entire as showed in Fig. 1). In Olivotto laboratory were performee, on one slide, histological analyses, while, in Battistelli laboratory were performed ultrastructural evaluation.

### Histological Analyses

Samples of labral tissue from all subjects were processed for histology after fixation in 4% formaldehyde and for the histological analysis, two/three sequential sections were scored for each case as previously described [12]. To asses labral tissue morphology and degradation evaluating proteoglycan/collagen content the sections were stained with hematoxylin–eosin (H&E) (Bioptica, Milano, Italy) and 0.25% Safranin-O/0.3% Fast Green (S–O-FG) (Sigma Aldrich, St Louis, MO) observed at 10 × magnification. The histological degeneration grade was assessed in all samples by a modified Pauli’s score as described in our previous study [17]. Briefly, Pauli’s microscopic grading system is validated to evaluate changes in the whole aging and OA menisci (the meniscus was divided in 3 areas: femoral and tibial side, inner border) [20].. We modified original Pauli’s score because the collection of FAI labral specimens is usually possible in only one area: the acetabular rim [17]. The grading system is composed of four categories (I–surface, II–cellularity, III–collagen organization/alignment and fiber organization and IV–matrix staining) as already reported [12, 17, 21]. The grades are defined as follows: grade 1, 0–2; grade 2, 3–5; grade 3, 6–9; and grade 4, 10–12. Grade 1 represents normal tissue, grade 2 and grade 3 indicate mild and moderate degeneration, respectively, and grade 4 represents severe degeneration [17]. Moreover, in samples obtained from MCDs and OA subjects it was possible to use the original Pauli’s score [20].

To evaluate calcification in labral samples, the histological sections were stained with 1.4% Alizarin Red prepared in distilled water and the pH was adjusted to pH 4.2 using 10% (v/v) ammonium hydroxide (Sigma Aldrich, St Louis, MO). Calcium crystal deposition score included four grades: 0, no deposition, from grade one to three depending on the size and number of deposits in the section [12, 20]. All images were captured with a Nikon Eclipse 90i microscope equipped with Nikon Imaging Software elements.

### Transmission Electron Microscopy

Samples were fixed with 2.5% glutaraldehyde in 0.1 M phosphate buffer for 3 h, incubated with 1% OsO_4_ in the same buffer for one hour, alcohol dehydrated, and embedded in araldite, as previously reported [22]. Thin sections were collected on 400 mesh nickel grids and stained with uranyl acetate and lead citrate. We make semithin section, 1 µm, for first evaluation of sample overview, and after that ultrathin section, 0.5 µm, for ultrastructural evaluation. The observations were carried out with a Philips CM 10 electron microscope at 80 kV [23]. A qualitative analysis of the ECM features was performed on labral tissues, as described by Olivotto et al. [24]. To measure the collagen fiber diameter, we carried out a careful analysis of 20 sections for each sample, recording observations in ten different fields for each section [25].

A qualitative analysis was performed to identify viable cells and cells during different types or steps of cell death known to occur in fibrochondrocytes [26].

## Statistical Analysis

Statistical analysis was performed with GraphPad Prism 6.0 statistical software (GraphPad Prism Software, Inc.). Continuous variables were expressed as median and IQR, while categorical data were expressed as raw numbers or percentages. Given the small sample size, non-parametric tests were expressed as. *P*-values less than 0.05 were considered significant.

## Results

### Extracellular Matrix Changes

Labrum was analyzed in patients affected by late OA and MCDs to evaluate geographical differences of the whole labral structure (Fig. 1). As shown in the figure each labral sample was cut perpendicular relative to the sagittal plane and different regions named as following were analyzed: A = anterior, B = middle and C = posterior region. In case of FAI patients, only small specimens of discarded tissue from the impingement area were available for analysis, thus not accounting for the features of the entire labrum.

**Fig. 2:**
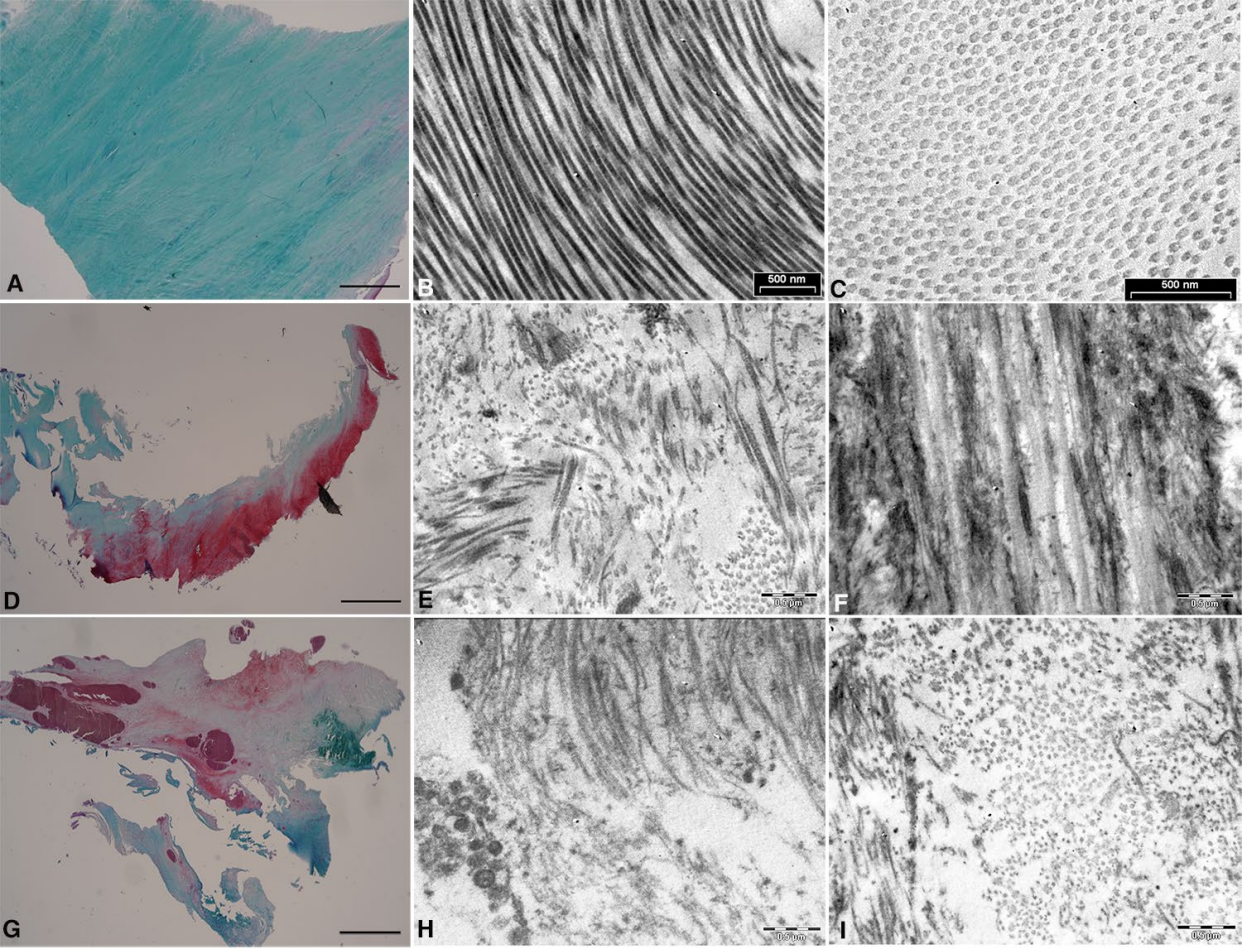
Labral tissue morphology. Labral samples collected from one representative multi-organ donor showed strong stain for Collagen and virtually no staining for proteoglycans observed by Safranin-O/Fast green staining (**A**). The collagen fibers appeared with homogenous distribution, regular size and orientation by transmission electron microscopy (TEM) (**B**–**C**). In samples from one representative patient with FAI (**D**–**F**) and with end-stage OA (**G** and **H**), there was a strong staining for proteoglycans and the TEM revealed collagen fibers with a structural disorganization of the ECM (Magnification: A, D, G = 2 x, bar 1000 μm; B, C, E, F, H, I = bar 0.5 µm)

The samples collected from the two youngest MCDs (17 and 35 years old, anterior region of the labrum, from 9:00 to 12:00), showed a strong intensity of Fast Green staining as shown in the figure (Fig. 2A). Fast Green is a specific histological staining for cartilage, a strong intensity of Fast Green staining shows the collagen fiber presence in the samples. In the third donor (median age 46 years), the ECM structure was less compact with some separations among collagen fibers and less intense Fast Green staining. In two patients out of three we also found the presence of proteoglycans staining by Safranin-O, which suggests loss of collagen fibers, in one region out of three analyzed in the same biopsy (Supplementary Fig. 1).

TEM analysis confirmed homogenous distribution and orientation of collagen fibers in all samples. The collagen fibers were aligned with a regular pattern and distribution in both longitudinal and transverse sections. The collagen fiber diameter varied from 70 to 80 nm (Fig. 2B, C ), as described in the literature for the standard collagen fibers (18). Periodic collagen organization, mainly collagen Type II, which is the major fibrillar component of the inner-zone, was observed. All patients with FAI showed normal tissue with signs of structural disorganization of collagen fibers in the superficial layers except in the older patient in which there was a mild degeneration throughout the thickness of the biopsy with the same features found in OA patients (Fig. 2D). This patient has also the worst KL grade among the FAI patients (grade 2) [27].

TEM observations showed that FAI patients had structural disorganization of the ECM, including loss of collagen fiber organization. The collagen fibers were disorganized and their diameter was heterogeneous within the same specimen, the collagen fibre distribution showed a diffuse disorganization and the fibre had a different diameter and in numerous fibre disappear typical period, probably because there was the presence of many immature fiber. The fiber diameter was between 45 and 55 nm (Fig. 2E, F). Among those patients, the 51-year-old patient showed the worst degeneration with a collagen fiber diameter between 30 and 45 nm (Fig. 2C, inset). In general, in patients with OA the labrum showed a reduction and structural disorganization of collagen fibers with strong prevalence of proteoglycans (Fig. 2G). As expected, at TEM observation OA patients showed a reduction of collagen fiber with a periodic organization and we observed numerous fibrils and proteoglycans that structurally replace collagen fibers with fiber diameter varying from 30 to 40 nm (Fig. 2H–L). The total histological degeneration grade assessed by modified Pauli’s score for all samples is described in Table 1. The components of the total labral degeneration score (original Pauli’s score) for MCDs and OA samples are described in Supplementary Table 2.

**Table 1:** Components of the total labral degeneration score and total histological degeneration grade (modified Pauli’s score)

Sample	Surface	Cellularity	Collagen organization	Saf-O-Fast green	Total degeneration score (0–12)
D#1	1	0	0	0	Grade = 1 (1)
D#2	0	0	0	0	Grade = 1 (0)
D#3	1	2	0	1	Grade = 2 (4)
D#4	1	2	1	1	Grade = 2 (5)
D#5	0	0	0	0	Grade = 1 (0)
FAI#1	2	0	0	0	Grade = 1 (2)
FAI #2	2	0	0	0	Grade = 1 (2)
FAI #3	3	1	0	0	Grade = 1 (4)
FAI #4	3	1	3	2	Grade = 2 (9)
FAI #5	2	0	0	0	Grade = 1 (2)
OA#1	2	1	2	3	Grade = 3 (8)
OA#2	3	3	3	3	Grade = 4 (12)
OA#3	3	2	2	3	Grade = 4 (10)
OA#4	3	2	3	3	Grade = 4 (11)
OA#5	3	2	3	3	Grade = 4 (11)

*D* donor; *FAI* femoroacetabular impingement; *OA* osteoarthritis; *Saf-O* safranin-O

### Calcium Deposits

**Fig. 3:**
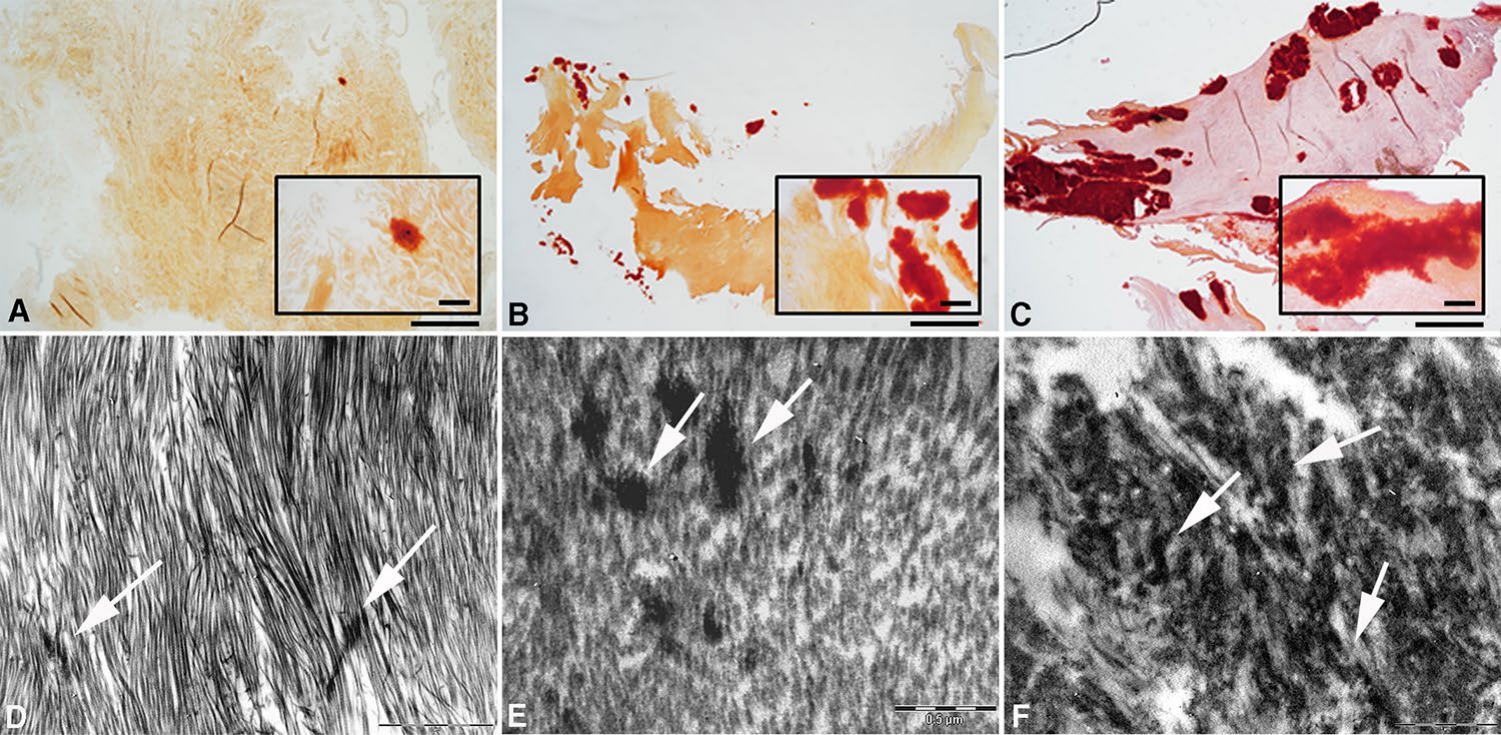
Calcium deposition. Labrum samples collected from one representative multi-organ donor showed small calcium deposits, evaluated by alizarin red (AR) staining (**A** and insert) and rarely observed with TEM (**D**). In FAI samples big calcium deposits were visible with AR (**B** and insert) also detected by TEM (**E**). Extensive calcified areas were observed in one representative OA patient (**C** and **F**) (White arrows = calcium deposits. Magnification panel A, B, C = 2x, bar 1000 μm; insert 10x; panel D = bar 2 µm, E = bar 0.5 µm, F = bar 1 µm)

The presence of calcium deposits were evaluated in the labral samples by alizarin red staining and TEM. Among those collected from MCDs, we observed only in the youngest donor (17 years old) the absence of calcium deposits, on the contrary rare calcium deposits were found in all sample (Fig. 3A–C). In general, in samples from FAI we observed the presence of large calcium deposits only in the older patient (Fig. 3B–E). As expected, we observed extensive calcified areas in labral samples of all subjects with OA (Fig. 3C–F).

### Cell Morphology

**Fig. 4:**
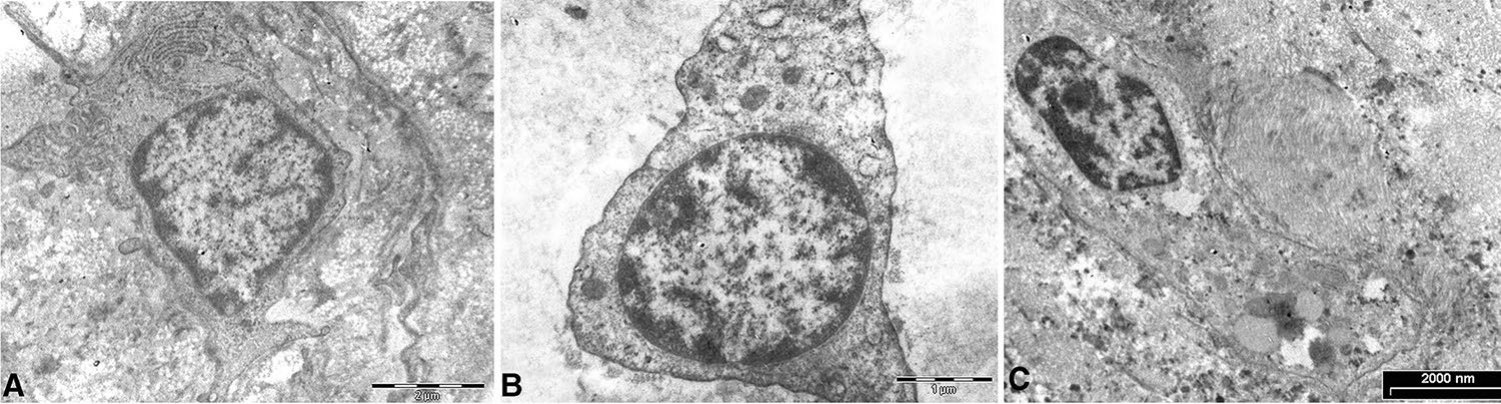
Cell morphology. In labrum from MCDs, the cells embedded in the ECM were round and showed a healthy morphology, consistent with viable and metabolically active cells. The nuclear chromatin appeared diffuse with small condensed areas near the nuclear membrane (**A**). In patient with FAI cells contained condensed nuclear chromatin, with a specific pattern, called ‘‘chondroptosis’’. Cytoplasmic vacuoles were present with scarcity of organelles (**B**). In the representative OA patient large areas of condensed chromatin (**C**) and vacuoles within the cytoplasm were observed (**C**). TEM A, C bar 2 µm, B bar 1 µm

The cells features were analyzed with TEM. In MCDs, labral samples showed fibrochondrocytes embedded in the ECM with rounded healthy morphology, and the nuclei exhibited diffuse chromatin with small condensed areas near the nuclear membrane (Fig. 4A). The cytoplasm contained a high amount of glycogen mitochondria were round and swollen and the rough endoplasmic reticulum was abundantly and well preserved. We observed small autophagic vacuoles. In patients with FAI, the chromatin showed small condensed areas similar to the chromatin in the multi-organ donor, whereas small rare vacuoles and autophagic vacuoles could be observed in the cytoplasm. In the older patients, the nuclear chromatin appeared to be more condensed compared to that in the MCDs. Chromatin margination and condensation with a specific pattern, termed by Roach et al. [28] as ‘‘chondroptosis’’, was present in all samples from patients with traumatic injury (Fig. 4B). In the cytoplasm we observed abundant vacuoles but sparse cytoplasmic organelles, swollen and emptied mitochondria embedded in a relatively empty matrix. Autophagic vacuoles, presumably due to oxidative stress, were also present in the cytoplasm. FAI and OA (Fig. 4C) samples had cytoplasmic membrane well preserved, intact in the phospholipid bilayer and few conserved mitochondria. Moreover, in subjects with OA (Fig. 4C) we observed large areas of condensed chromatin and vacuoles within the cytoplasm. In patients with FAI and OA appeared a general loss of cytoplasmic organelles.

### Clinical Data

Based on the radiographic KL grading system for OA severity [27] with grade 0 signifying no presence of OA and grade 4 signifying severe OA, four FAI patients out of five showed a grade one of OA which means a possible joint space narrowing and osteophyte formation. The older patients presented definite osteophyte formation with possible joint space narrowing (KL grade two). In line with Kierkegaard et al. observation, as the first clinically relevant improvement in hip pain was observed at three to six months after hip arthroscopy [29], we evaluated the improvement of symptoms and functional limitations of the hip measured with the Hip Injury and Osteoarthritis Outcome Score (HOOS) total validated in Italian language (http://www.koos.nu/hoositalian.pdf) at 6 months after surgery. The interval score ranges from 0 to 100 where 0 indicating the worst possible hip symptoms and 100 indicating no hip symptoms.

We found that all patients showed a statistically significant improvement (23% of increase, p = 0.009) (Supplementary Fig. 2). We already found a general improvement of FAI patients after surgery in our previous published study with a higher number of patients [12].

## Discussion

In the present study we found degenerative changes of the ECM and fibrochondrocytes damage in labral tissue collected from patients with FAI similar to the OA samples. In particular, we observed that the ECM of all FAI patients presented structural disorganization and, at the ultrastructural level, a thinning of the collagen fibers. As already demonstrates by Tschaikowsky et al. the collagen network exhibits changes in early OA by collagen fiber thinning [30] [31].

This degeneration, apparently, is not completely explained by ageing, since the mean age of patients with FAI was similar to MCDs. Despite some initial separation and loss of the collagen fibers was observed also in older MCDs, none of them presented with structural disorganization of the superficial layer of the labrum. It suggests that the chronic mechanical stress induced by the FAI mechanism, accelerates the labral deterioration and contributes to labral tears and symptoms onset. FAI also leads to chondropathies that cause pain and limited functional activities affecting activities of daily living ending with OA [32]. Moreover, the worst labral degeneration, with ECM loss, strong prevalence of proteoglycans, and large calcium deposits was observed in the older FAI patient. These microscopic features were similar to those of OA patients, enhancing the theory that there is a continuum between FAI and hip OA as we have already demonstrated in our previous study [12]. Calcium crystal deposition is common in OA acetabular labrum [14], also labral calcifications are present in patients with FAI [12, 33]. It has been proposed that labral calcifications may contribute to the onset of FAI symptoms, exacerbating pain (5, 28).

TEM analysis demonstrated that older FAI patients showed similar features to the OA subjects. Our findings suggest that the mechanical stress caused by the impingement can activate a process of labral degeneration and, ultimately, OA onset. Hip osteoarthritis is a disease process that impacts the aging population [34] and, currently considered a whole-joint disorder involving all of the joint tissues [35], it is the result of degeneration of the articular cartilage, underlying bone and soft tissue structures.

As we already showed in our previous study in the knee, meniscal biopsies collected from patients with meniscal tear showed similar features to those in OA patients (even though they were about 30 years younger). In contrast to meniscal samples from OA patients, the surface of those collected from patients with meniscal tear was often intact and there were distinct changes in ECM organization. This pattern is a typical age-related meniscal change in knees without history of major trauma. The release of inflammatory mediators in the joint space may trigger or accelerate the degenerative processes in the joint tissues [17].

We recently demonstrated that patients with FAI present with low-grade synovitis and perivascular mononuclear cell infiltration of the synovium associated with labral degeneration cartilage damage ageing and OA progression. It is possible that the substances released by the frayed labrum, could activate the synovium, transforming a mechanical stress in a chronic inflammatory process [36].

There are some limitations in our study. The small sample size does not allow generalization of our findings. Moreover, in FAI patients only small specimens of discarded labral tissue could have been analyzed, does not account for the features of the entire labrum. Despite no relevant clinical history of hip disease reported in MCDs, and no macroscopic appearance of OA or FAI deformities observed during the tissue procurement, we cannot be sure that the MCDs were free from hip symptoms or radiographic aspects of FAI. In FAI surgery, the preservation of labral function is the final goal and, the small specimens obtained from FAI surgery made the histological analysis challenging making some patients eligible for the study not included. Moreover, the availability of samples from MCDs is rare because the collected tissues are used for transplantation aims. On the other hand, although the rate of discarded joint tissues is very low, only in this case those precious tissues may be used for validation protocols or surgical/transplant procedure optimization. It is possible to obtain fresh samples from MCDs only in case they were not used during the operation after assignment. Further research involving larger numbers of subjects per group would enhance the significance of data.

In conclusion based on our microscopic observation, a careful examination of preoperative joint status not only based on clinical history, symptoms and pain but as well with imaging to evaluate the joint tissue condition, might be an important aspect of patient selection for a successful and stable surgical procedure.

## Supplementary Information

Below is the link to the electronic supplementary material.

Supplementary file1 (JPG 382 KB)—Labral tissue samples from MCDs. For each panel a section of anterior, middle and posterior region of the entire sample stained with Safranin O-Fast green. **A** 17 years old patient; **B** 35 years old patient; **C** 42 years old patient; **D** 47 years old patient, **E** 48 years old patient (Magnification 2x, bar 1000 μm)

Supplementary file2 (JPG 369 KB)—Comparison between pre and post-operatively HOOS at 6 months after arthroscopy. The five panels from the left clockwise show the HOOS subscales: symptoms, pain, function, activity limitations in sport and hip related quality of life (QOL). The last panel on the bottom right shows the pre and post-operatively total HOOS (*p < 0.05; **p < 0.01). Scale 20–100, worst to best.

Supplementary file3 (DOCX 13 KB)

Supplementary file4 (DOCX 14 KB)
