# Supplementary figures and images for: Hip Labral Morphological Changes in Patients with Femoroacetabular Impingement Speed Up the Onset of Early Osteoarthritis
Source: Calcif Tissue Int. 2023 Mar 22;112(6):666–74. doi: 10.1007/s00223-023-01076-1 (PMC10199105; doi:10.1007/s00223-023-01076-1)

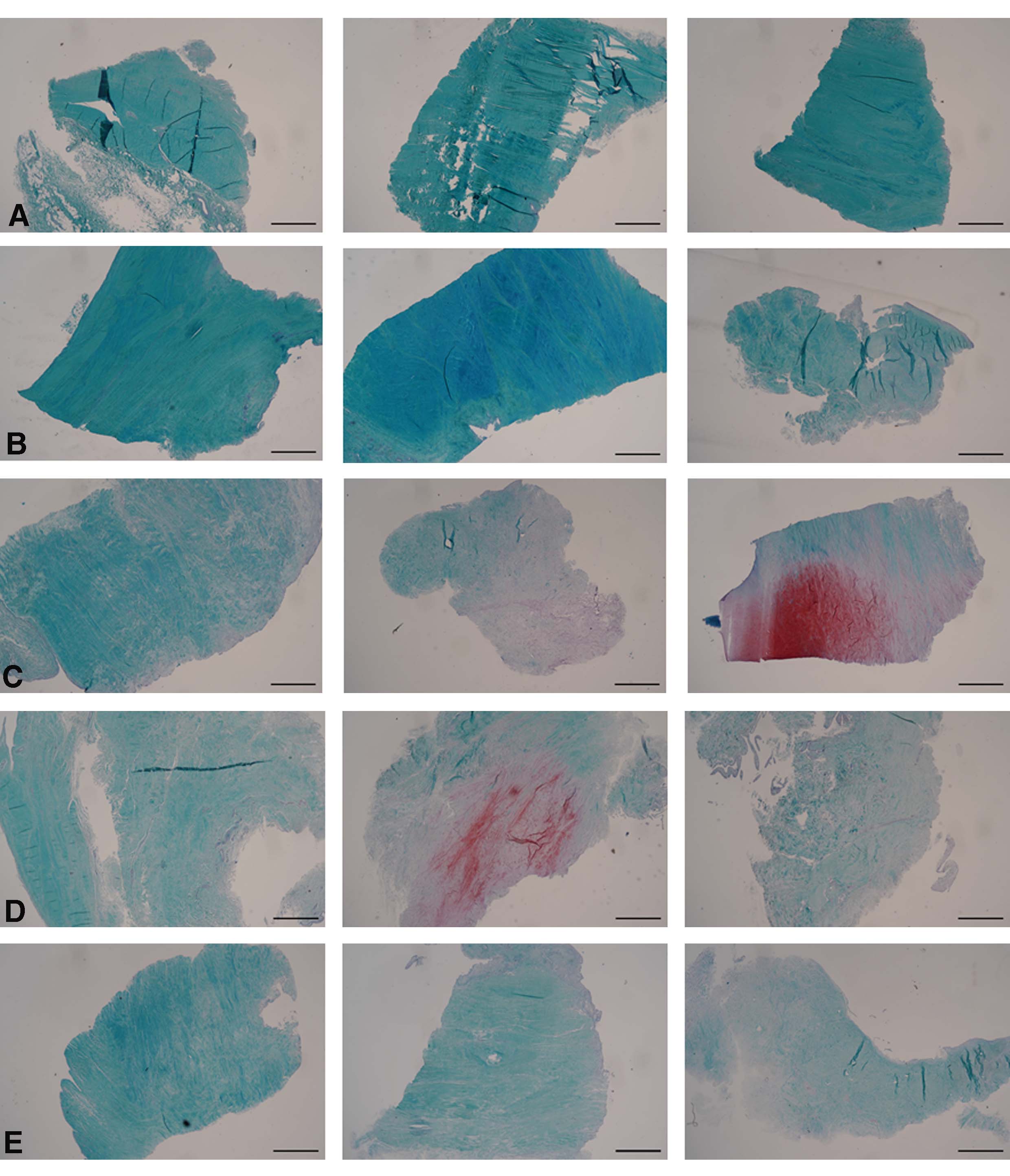

Supplement: Supplementary file 1 — Supplementary file1 (JPG 382 KB)—Labral tissue samples from MCDs. For each panel a section of anterior, middle and posterior region of the entire sample stained with Safranin O-Fast green. A 17 years old patient; B 35 years old patient; C 42 years old patient; D 47 years old patient, E 48 years old patient (Magnification 2x, bar 1000 μm) [file 223_2023_1076_MOESM1_ESM.jpg]

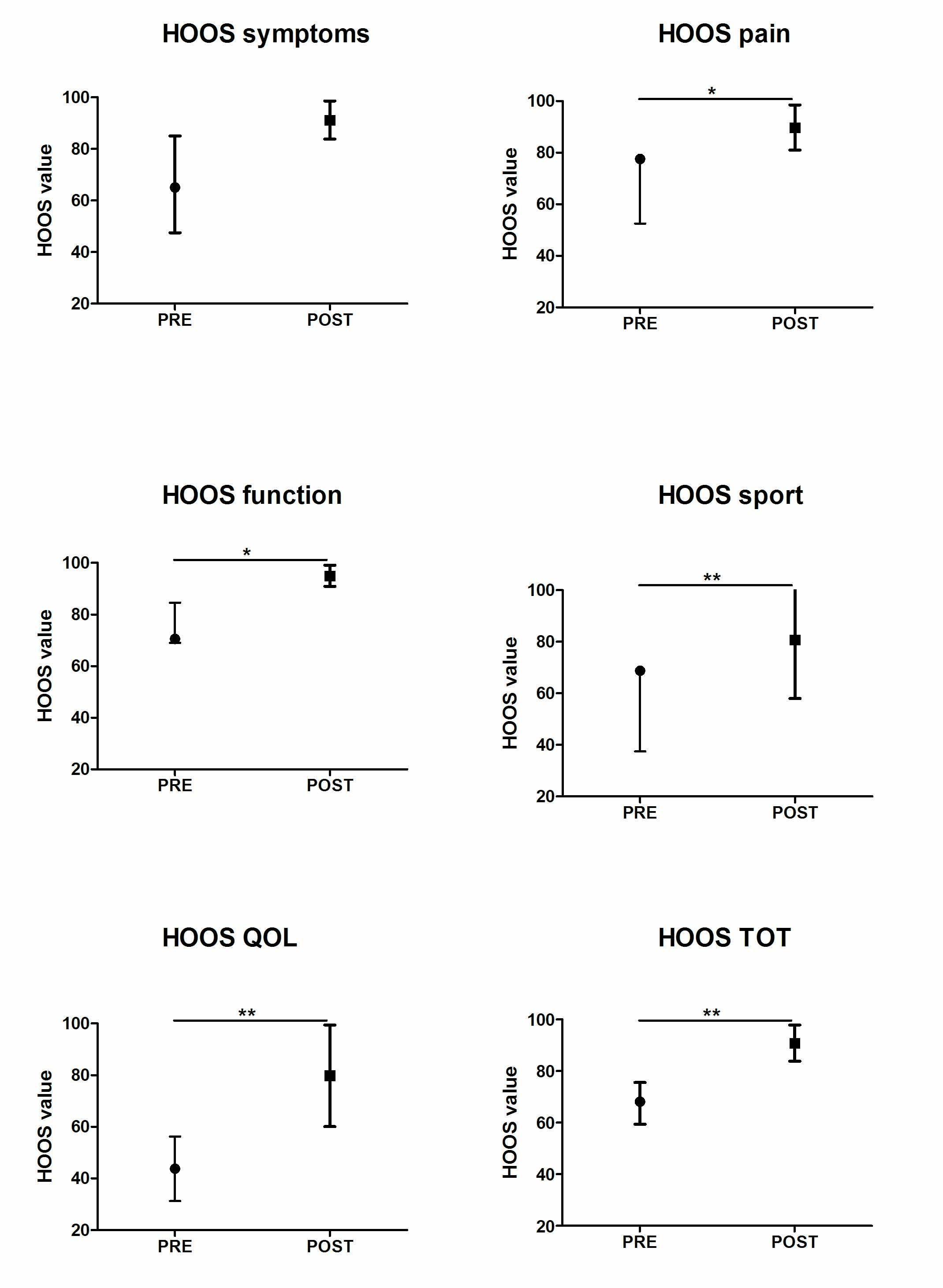

Supplement: Supplementary file 2 — Supplementary file2 (JPG 369 KB)—Comparison between pre and post-operatively HOOS at 6 months after arthroscopy. The five panels from the left clockwise show the HOOS subscales: symptoms, pain, function, activity limitations in sport and hip related quality of life (QOL). The last panel on the bottom right shows the pre and post-operatively total HOOS (*p < 0.05; **p < 0.01). Scale 20–100, worst to best. [file 223_2023_1076_MOESM2_ESM.jpg]
