# Supplementary material for: Hip Labral Morphological Changes in Patients with Femoroacetabular Impingement Speed Up the Onset of Early Osteoarthritis
Source: Calcif Tissue Int. 2023 Mar 22;112(6):666–74. doi: 10.1007/s00223-023-01076-1 (PMC10199105; doi:10.1007/s00223-023-01076-1)
Supplement: Supplementary file 3 — Supplementary file3 (DOCX 13 KB) [file 223_2023_1076_MOESM3_ESM.docx]

| **Supplementary table 1.**  **Clinical characteristics of patients with FAI (N = 5)** | |
| --- | --- |
| Labral lesion, number | 5 |
| Kellgren-Lawrence^#^ 0-1/2/3/4 grade, number | 4/1/0/0 |
| Acetabular chondropathy*, 1/2/3/4 grade, number | 1/3/1 |
| Femoral chondropathy*, 1/2/3/4 grade, number | 2/2/1 |
| Median HOOS^§^ total preoperative (IQR) | 67.62 (57.5-75.6) |
| Median HOOS total postoperative (IQR) | 90.84 (80.6-97.5) |
| **Clinical characteristics of patients with OA (N = 5)** |  |
| Labral lesion, number | 5 |
| Kellgren-Lawrence^#^ 1/2/3/4 grade, number | 0/0/0/5 |
| Acetabular chondropathy*, 1/2/3/4 grade, number | 0/0/0/5 |
| Femoral chondropathy*, 1/2/3/4 grade, number | 0/0/0/5 |

^#^Kellgren-Lawrence (KL) grading system for OA severity; *Outerbridge score for cartilage lesion

^§^Pre- and post-operative symptoms (6 months) were evaluated using the Hip Injury and Osteoarthritis Outcome Score (HOOS) total validated in Italian language (http://www.koos.nu/hoositalian.pdf).
