# Supplementary material for: Hip Labral Morphological Changes in Patients with Femoroacetabular Impingement Speed Up the Onset of Early Osteoarthritis
Source: Calcif Tissue Int. 2023 Mar 22;112(6):666–74. doi: 10.1007/s00223-023-01076-1 (PMC10199105; doi:10.1007/s00223-023-01076-1)
Supplement: Supplementary file 4 — Supplementary file4 (DOCX 14 KB) [file 223_2023_1076_MOESM4_ESM.docx]

| **Supplementary table 1.** Components of the total labral degeneration score and total histological degeneration grade (original Pauli’s score) | | | | | | |
| --- | --- | --- | --- | --- | --- | --- |
| **Sample** | **Zone** | **Surface** | **Cellularity** | **Collagen**  **Organization** | **Saf-O-Fast Green** | **Total Degeneration score (0-18)** |
| **D#1** | A | 1 | 0 | 0 | 0 | **Grade = 1 (3)** |
|  | B | 1 |  |  |  |  |
|  | C | 1 |  |  |  |  |
| **D#2** | A1 | 1 | 0 | 0 | 1 | **Grade = 1 (2)** |
|  | B1 | 0 |  |  |  |  |
|  | C1 | 0 |  |  |  |  |
| **D#2** | A2 | 0 | 0 | 0 | 0 | **Grade = 1 (0)** |
|  | B2 | 0 |  |  |  |  |
|  | C2 | 0 |  |  |  |  |
| **D#3** | A | 1 | 2 | 0 | 2 | **Grade = 2 (8)** |
|  | B | 1 |  |  |  |  |
|  | C | 1 |  |  |  |  |
| **D#4** | A | 1 | 2 | 1 | 1 | **Grade = 2 (7)** |
|  | B | 1 |  |  |  |  |
|  | C | 1 |  |  |  |  |
| **D#5** | A | 0 | 0 | 0 | 0 | **Grade = 1 (0)** |
|  | B | 0 |  |  |  |  |
|  | C | 0 |  |  |  |  |
| **OA#1** | A | 2 | 2 | 2 | 3 | **Grade = 4 (15)** |
|  | B | 3 |  |  |  |  |
|  | C | 3 |  |  |  |  |
| **OA#2** | A | 3 | 3 | 3 | 3 | **Grade = 4 (15)** |
|  | B | 3 |  |  |  |  |

D = donor; FAI = femoroacetabular impingement; OA = osteoarthritis; Saf-O = safranin-O
